# Efecto de las medidas preventivas durante la pandemia: más allá del SARS CoV-2

**Published:** 2021-10-15

**Authors:** Aura Lucía Leal-Castro

**Affiliations:** 1 Departamento de Microbiología, Facultad de Medicina, Universidad Nacional de Colombia, Bogotá, D.C., Colombia allealc@unal.edu.co Universidad Nacional de Colombia Departamento de Microbiología Facultad de Medicina Universidad Nacional de Colombia BogotáD.C. Colombia allealc@unal.edu.co

La situación que atraviesa actualmente el mundo con la pandemia de la COVID-19 tiene un impacto global sin precedentes sobre la morbimortalidad, especialmente en poblaciones vulnerables. Si bien todos los esfuerzos desplegados a lo largo de más de un año a nivel local, nacional e internacional estaban orientados a disminuir la transmisión del SARS CoV-2, las medidas sanitarias han tenido efecto en aquellos agentes patógenos cuyos modelos de transmisión son similares a los de este. La interrupción de la transmisión “persona a persona” es la razón más probable para explicar la disminución en la circulación de los virus respiratorios estacionales y en la presentación de infecciones bacterianas respiratorias por *Streptococcus pneumoniae*, *Haemophilus influenzae* e, incluso, *Neisseria meningitidis*, cuya vía de transmisión son las gotas. Además, la medida básica de prevención, es decir, el lavado de manos, tendría un impacto profundo, no solo en el comportamiento humano rutinario, sino en la disminución de infecciones como la enfermedad diarreica aguda ^1^.

En un comienzo, preocupaba el efecto potencial de la pandemia en la carga de infección respiratoria en algunos países, debido a la circulación concomitante de los virus respiratorios estacionales que se esperaba, según las proyecciones de los sistemas de vigilancia establecidos. Sin embargo, en la actualidad se reconoce que las medidas instauradas, como las restricciones en desplazamientos, el confinamiento, el uso de mascarillas, el distanciamiento y la higiene de manos, han tenido efecto sobre la circulación de algunos de estos virus y las infecciones que causan ^2^. Durante la pandemia ha aumentado considerablemente el número de estudios que generan evidencia científica a favor de dichas medidas para el control de la transmisión, por ejemplo, aquellos sobre el impacto del distanciamiento social y el efecto real de los tapabocas o barbijos ^3^.

En el 2020, la detección de virus respiratorios, como el de la influenza o el respiratorio sincitial, registró una importante disminución comparada con la del 2019, según los informes de los sistemas de vigilancia de países en todo el mundo. Algunos sistemas incluso informaron la “ausencia efectiva” de la epidemia anual, con un especial impacto en la población pediátrica apreciable en la reducción significativa de las tasas de ingreso a las unidades de cuidado intensivo por infecciones respiratorias graves ^4^. Colombia no ha sido ajena a este fenómeno y, en el 2020, los reportes de casos de infección respiratoria disminuyeron el 38 %, en tanto que no se recuperaron virus como el de influenza, el respiratorio sincitial, los adenovirus o los metapneumovirus durante las semanas epidemiológicas 27 a 37, comportamiento inusual y muy diferente al de años anteriores cuando, para esa misma época, se detectaban los picos de la mayoría de los virus respiratorios en nuestro país ^5^.

En las infecciones bacterianas como la meningitis meningocócica, también se reportó una disminución marcada en el mundo, por ejemplo, el sistema de vigilancia del Reino Unido registró una reducción del 76 % en el 2020 ^6^. En Colombia, se presentó una reducción global del 48 % en los casos de meningitis bacteriana aguda por *S. pneumoniae, H. influenzae y N. meningitidis*, así como en otras infecciones como la tosferina, con una disminución del 78 % en la notificación de casos ^7^^,^^8^, y la enfermedad diarreica aguda o la hepatitis A, que disminuyeron 45 y 78 %, respectivamente, en comparación con el año inmediatamente anterior ^9^^,^^10^.

El impacto de las medidas instauradas en la pandemia va incluso mas allá de las infecciones virales y bacterianas. Así lo demuestra la disminución significativa de la prevalencia de pediculosis en Latinoamérica como consecuencia de la restricción en la movilidad de los niños y la inasistencia a los centros educativos ^11^.

Algunos autores han señalado que el impacto observado podría considerarse una “consecuencia positiva no esperada” de las medidas, aunque la magnitud y la duración de dicho efecto variaría, como en el caso de los virus respiratorios, según el tipo de agente ^3^^,^^4^.

De todas maneras, el efecto de las medidas en la comunidad justificaría adoptarlas en el futuro para disminuir la transmisión de muchos de estos agentes patógenos. Sin embargo, no se ha observado el mismo impacto en otras situaciones, como la prevención de infecciones asociadas con la atención en salud, pues, aunque hay una mayor visibilidad de prácticas como la higiene de manos, las medidas de aislamiento o el uso de elementos de protección individual, múltiples informes coinciden en que este tipo de infecciones asociadas con bacterias resistentes aumentó durante los picos de ingreso de pacientes con COVID-19. En este sentido, se podría afirmar que las tasas de dichas infecciones se han visto influenciadas negativamente por la reacción frente a la pandemia ^12^.

Más allá de los datos observados, hay un aspecto fundamental que llama a la reflexión: la pandemia y las medidas para combatirla han ejercido un efecto sobre el comportamiento y las dinámicas de la prestación de los servicios de salud, su acceso, la capacidad para hacer pruebas diagnósticas, los sistemas de notificación cuya base es la generación de diagnósticos y que se han visto influenciados por múltiples factores, incluidas la carga de trabajo y la priorización, tanto de los prestadores como de las autoridades de salud. Por lo tanto, el reto radica en determinar si la disminución en el reporte de casos de infecciones realmente corresponde a una menor carga de la enfermedad por reducción de la transmisión, o si se debe al subregistro en la vigilancia rutinaria ^13^.

Tal vez la principal lección aprendida hasta el momento es la necesidad de establecer el equilibrio entre las demandas de atención de la pandemia, y el mantenimiento de los procesos rutinarios de vigilancia, prevención y control de enfermedades transmisibles y no transmisibles. Los sistemas de salud y las políticas públicas deben adelantar una evaluación continua de las medidas instauradas durante la pandemia y su posible impacto en otros ámbitos de la salud.

Nos estamos enfrentando a grandes retos globales que obligan a “mirar por fuera de la caja”, es decir, a adoptar una visión centrada, no solamente en el impacto de las medidas contra la transmisión del SARS CoV-2, sino en la posibilidad de transferir algunos de sus principios al control de otras infecciones. Además, los cambios observados en la esfera de la epidemiología se deben a múltiples causas y el reto, sin duda, será evaluar el impacto que la reacción a la pandemia ha generado en los sistemas de vigilancia y, eventualmente, instaurar estrategias para fortalecerlos en un futuro inmediato.
